# Supplementary material for: Prepregnancy BMI, gestational weight gain and offspring caries experience: Avon longitudinal study of parents and children
Source: PLoS One. 2022 Mar 31;17(3):e0266247. doi: 10.1371/journal.pone.0266247 (PMC8970488; doi:10.1371/journal.pone.0266247)

**Supporting information**

**S1Table**. IOM-Recommended levels of GWG according to prepregnancy BMI categories [30].

| **Pre-pregnancy BMI** | **Recommended range of absolute weight gain, kg** |
| --- | --- |
| Underweight (<18.5kg/m^2^) | 12.5-18 |
| Normal (18.5-24.9kg/m^2^) | 11.5-16 |
| Overweight (25-29.9kg/m^2^) | 7-11.5 |
| Obese (≥30kg/m^2^) | 5-9 |
| IOM-Institute of Medicine |  |

**S2Table. Proportion of missing observations for imputed variables**

| **Variables** | **Missing proportion** |
| --- | --- |
| #decayed, missing and filled teeth at 31, 41 and 61 months | 19%; 24%; 34% |
| Absolute weight gain | 6% |
| Pregnancy weight and height | 10%; 7% |
| Prenatal smoking | 4% |
| Moves in last 5 years, maternal race, and education | 4%; 5%; 5% |
| Gestational diabetes, gestational hypertension, method of delivery | 0.07%; 0.3%; 0.4% |

**S1Fig.** Directed Acyclic graph for gestational weight gain and early childhood caries
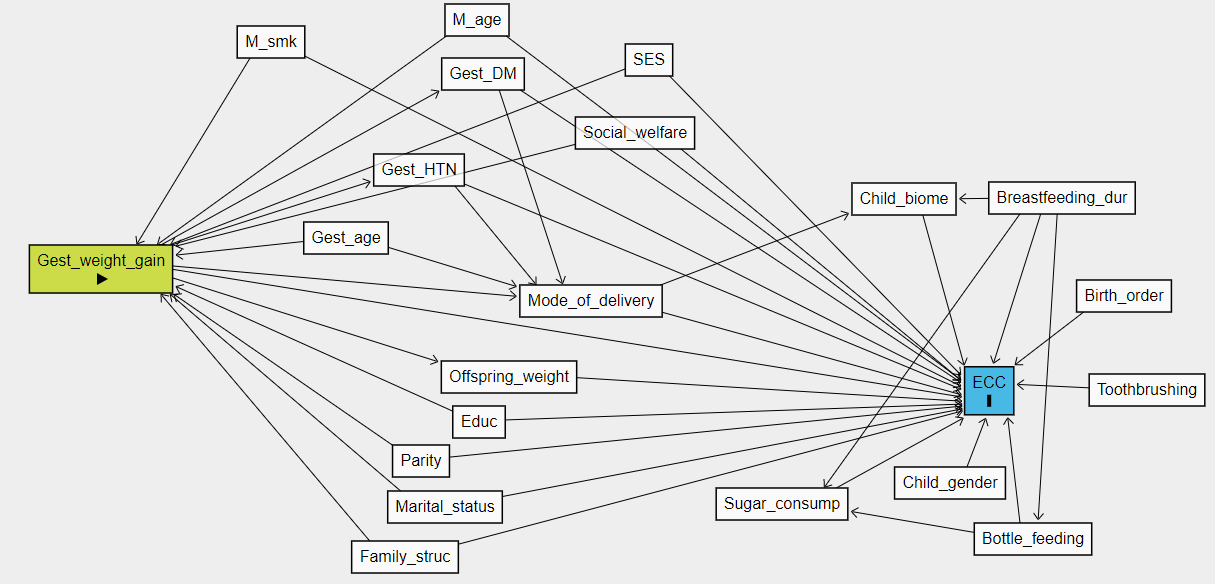

Supplement: S1 File — (DOCX) [file pone.0266247.s001.docx]
