# Supplementary material for: Prepregnancy BMI, gestational weight gain and offspring caries experience: Avon longitudinal study of parents and children
Source: PLoS One. 2022 Mar 31;17(3):e0266247. doi: 10.1371/journal.pone.0266247 (PMC8970488; doi:10.1371/journal.pone.0266247)
Supplement: Attachment — Submitted filename: Response to Reviewers2.docx [file pone.0266247.s003.docx]

The authors thank the reviewers for the second round of review of our manuscript: “PONE-D-21-29637R1, Prepregnancy BMI, gestational weight gain and offspring caries experience: Avon Longitudinal Study of Parents and Children”. Our response to reviewers’ comments appears below and in track changes in the actual manuscript draft. Please note that the latest iteration of track changes is only for the reviewers remaining concerns and does not include track changes from the previous submission which has been disabled.

**Comments to the Author**

Additional Editor Comments (if provided):

Dear authors,
multiple comments received in the first review round have not been addressed, eg amendment of the Discussion section and expansion on the Limitations of the study (Reviewer 1, comment 10). Please respond to these comments and make appropriate changes to the Discussion section, as recommended.

Authors’ response: Pasted below are the specific comments related to the discussion section from the previous review and our response to those comments

Discussion
1. Many of the factors were not discussed in the manuscript, as well as the many limitations of the study. The discussion is very limited and insufficient to support the findings of the study.

Response: We discussed the findings of our analysis of whether pre-pregnancy BMI, and pregnancy weight are associated with offspring caries experience. Again, there is not a lot of literature out there to the authors knowledge to compare and contrast our findings with and we avoided making speculations beyond what an epidemiological study can tell us.

Updated response: Please note that the association between adjustment covariates and the outcome of ECC was not the primary purpose of this study. Because we have not accounted for the confounding structure between those adjustment covariates and ECC, discussing their individual association from a model of pre-pregnancy BMI, and pregnancy weight and ECC is fallacious (please refer to the paper by Westreich and Greenland 2013 for detail of this fallacy)

2. One of the main findings were the fact that less than recommended GWG was associated with higher caries. How do the authors explain this finding?

Response: Yes, the magnitude of the effect was small and not statistically significant

Updated response: The following text has been added to the discussion

“The weak association we found for prepregnancy BMI and offspring caries may thus, be a reflection of prepregnancy BMI being a more distal risk factor in comparison to obesity in the offspring.”

“Of note, our study assessed weight gain during pregnancy according to levels recommended by the IOM for different prepregnancy BMI groups. We found gestational weight gain less and more that recommended was associated with greater offspring caries experience risk, although not statistically significant.”

Reviewers' comments:

Reviewer's Responses to Questions

**Comments to the Author**

1. If the authors have adequately addressed your comments raised in a previous round of review and you feel that this manuscript is now acceptable for publication, you may indicate that here to bypass the “Comments to the Author” section, enter your conflict of interest statement in the “Confidential to Editor” section, and submit your "Accept" recommendation.

Reviewer #1: (No Response)

2. Is the manuscript technically sound, and do the data support the conclusions?

The manuscript must describe a technically sound piece of scientific research with data that supports the conclusions. Experiments must have been conducted rigorously, with appropriate controls, replication, and sample sizes. The conclusions must be drawn appropriately based on the data presented.

Reviewer #1: No

3. Has the statistical analysis been performed appropriately and rigorously?

Reviewer #1: Yes

4. Have the authors made all data underlying the findings in their manuscript fully available?

The [PLOS Data policy](http://www.plosone.org/static/policies.action#sharing) requires authors to make all data underlying the findings described in their manuscript fully available without restriction, with rare exception (please refer to the Data Availability Statement in the manuscript PDF file). The data should be provided as part of the manuscript or its supporting information, or deposited to a public repository. For example, in addition to summary statistics, the data points behind means, medians and variance measures should be available. If there are restrictions on publicly sharing data—e.g. participant privacy or use of data from a third party—those must be specified.

Reviewer #1: Yes

5. Is the manuscript presented in an intelligible fashion and written in standard English?

PLOS ONE does not copyedit accepted manuscripts, so the language in submitted articles must be clear, correct, and unambiguous. Any typographical or grammatical errors should be corrected at revision, so please note any specific errors here.

Reviewer #1: Yes

6. Review Comments to the Author

Please use the space provided to explain your answers to the questions above. You may also include additional comments for the author, including concerns about dual publication, research ethics, or publication ethics. (Please upload your review as an attachment if it exceeds 20,000 characters)

Reviewer #1: Some corrections were made, however the authors did not response to the major limitations in the methodology and for this reason I do not think it is suitable for publication.

Response: The authors do not understand the specific concern that the reviewer has with our manuscript or the limitations with the methodology that we have not addressed. Nevertheless, we have expanded the discussion section and the limitation section in the revised manuscript.

We also want to point that the association between adjustment covariates and the outcome of ECC was not the primary purpose of this study. Because we have not accounted for the confounding structure between those adjustment covariates and ECC, discussing their individual association from a model of pre-pregnancy BMI, and pregnancy weight and ECC is fallacious (please refer to the paper by Westreich and Greenland 2013 for detail of this fallacy)

Furthermore, the following text has been added to the discussion

“The weak association we found for prepregnancy BMI and offspring caries may thus, be a reflection of prepregnancy BMI being a more distal risk factor in comparison to obesity in the offspring.”

“Of note, our study assessed weight gain during pregnancy according to levels recommended by the IOM for different prepregnancy BMI groups. We found gestational weight gain less and more that recommended was associated with greater offspring caries experience risk, although not statistically significant.”

7. PLOS authors have the option to publish the peer review history of their article ([what does this mean?](https://journals.plos.org/plosone/s/editorial-and-peer-review-process#loc-peer-review-history)). If published, this will include your full peer review and any attached files.


If you choose “no”, your identity will remain anonymous but your review may still be made public.


**Do you want your identity to be public for this peer review?** For information about this choice, including consent withdrawal, please see our [Privacy Policy](https://www.plos.org/privacy-policy).

Reviewer #1: No
